# ACE2 trials suggest remodeling, not dilation, as primary therapeutic effect

**DOI:** 10.1002/pul2.12022

**Published:** 2022-01-20

**Authors:** James West

**Affiliations:** ^1^ Vanderbilt University Nashville Tennessee USA

Angiotensin‐converting enzyme 2 (ACE2) has looked like a promising intervention for pulmonary arterial hypertension (PAH) for more than 10 years.^1^ Its activity is reduced in human PAH,^2^ and in preclinical studies, it was capable of preventing or reversing pulmonary hypertension in monocrotaline,^3^ hypoxic,^4^ and BMPR2 mutant^5^ rodent models.

ACE2 is an enzyme that cleaves the last amino acid from angiotensin II (AngII) to make Ang(1–7). It thus both decreases signaling through the AngII receptors, as well as, at least theoretically, increasing signaling through the Mas receptor, for which Ang(1–7) is the presumed ligand (although there is some conflicting information on this). Both of these functions are potentially beneficial in the context of PAH, with the potential for direct regulation of tone through angiotensin receptor blockers (ARBs),^6^ and regulation of remodeling through Ang(1–7).

Although ARBs did not appear effective in direct trials,^7^, ^8^ a recent retrospective study of a large cohort (24,221 patients) of pulmonary hypertension patients (of all etiologies) with and without ace inhibitors or ARBs found that those on these therapies were protected against mortality, with a hazard ratio of about 0.8.^9^ This discrepancy is probably a result of the effect being somewhat subtle. In contrast to ARBs, the effect of Ang(1–7), the enzymatic product of ACE2, is presumed to work by signaling through binding and activating signaling through the Mas1 receptor. This regulates Rac1 activation, with effects on angiogenesis and vascular permeability.^10^

This mass of preclinical data has led to small trials in human PAH patients; a trial of five patients at Vanderbilt,^2^ and now reported in this issue, Simon et al. conduct a somewhat larger dose‐escalation trial. Although the primary outcome was disappointing—no acute vasodilation—the report includes a wealth of detail on each patient and cohort, in the supplemental tables, which help us understand the current status of ACE2 as a therapy, particularly when compared to the earlier trial.

Both trials found ACE2 were safe and had the expected effects on AngII and Ang(1–7), but while the earlier trial found a modest signal for an acute reduction in pulmonary vascular resistance (PVR), the current trial did not. Both trials were single dose, with 4 h of hemodynamic data following that dose. The earlier trial used 0.2 and 0.4 mg/kg; the new trial used 0.1, 0.2, 0.4, and 0.8 mg/kg. All five patients in the earlier trial were idiopathic or heritable; the patients in the newer trial were these plus collagen‐vascular disease‐associated, particularly in the highest dose cohort (5/8). The most striking difference in the patient cohort between the two trials, though, was concomitant medications. Three of five patients in the earlier trial—the patients with the strongest reduction in PVR—were also on parenteral prostacyclin. None of the patients in the two highest treatment groups in the new study, and only 3 of 23 in the whole study used epoprostenol.

Combining these data with the results of earlier trials, in which ARBs were at best moderately effective, leads one to wonder whether the acute vasodilatory effects of ACE2 seen in the first trial were not because of the effect of ACE2 on the angiotensin receptors, the putative mechanism for acute dilation, but rather because of the effect of ACE2 on the Mas1 receptor, which might have repaired Rac1 pathway activity sufficiently to enable the concomitant epoprostenol's strong vasodilatory effect.

The earlier trial also included a number of endpoints related to Mas1 signaling, including a demonstrated reduction in reactive lipids, an increase in superoxide dismutase 2, and a reduction in several cytokines. Unfortunately, the newer trial did not test targets beyond BNP and NO (no change), and so the potential effect on Mas1 targets was not tested.

In sum, the trials in patients tell us three important things:

(1) Recombinant ACE2, at doses that strongly modulate angiotensin and Ang(1–7), is safe.
(2) ACE2 probably does not have an acute vasodilatory effect, at least independent of epoprostenol.
(3) ACE2—does—appear to impact the Mas1 pathway, which in the animal models, drove efficacy. Unfortunately, to be therapeutically effective, that requires long‐term treatment.

The big problem with ACE2, then, is the problem of manufacture; right now, enzymatically active ACE2 can only be made in adherent mammalian cells, which makes it incredibly difficult and expensive to make. In addition, ACE2 delivery currently requires an IV infusion of a short‐lived drug. The promise of ACE2 as a therapy for PAH will require either an improved method of manufacture, or an alternate means of activating the Mas receptor, and preferably an improved delivery mechanism.

ACE2 remains a promising therapeutic for pulmonary hypertension, but results, as presented both in earlier literature and in the trial in this issue, suggest that its strength will not be acute dilation, but rather impact on remodeling.
